# Effects of Ultrasound-Assisted Emulsification on the Emulsifying and Rheological Properties of Myofibrillar Protein Stabilized Pork Fat Emulsions

**DOI:** 10.3390/foods10061201

**Published:** 2021-05-26

**Authors:** Lei Zhou, Jian Zhang, Yantao Yin, Wangang Zhang, Yuling Yang

**Affiliations:** 1 Key Laboratory of Meat Processing and Quality Control, Ministry of Education China, Jiangsu Collaborative Innovation Center of Meat Production and Processing, Quality and Safety Control, College of Food Science and Technology, Nanjing Agricultural University, Nanjing 210095, China; judgeaie@163.com (L.Z.); 2019208029@njau.edu.cn (J.Z.); yantaoyin@126.com (Y.Y.); 2 College of Food Science and Engineering, Nanjing University of Finance and Economics, Nanjing 210046, China

**Keywords:** emulsified meat products, sonication, meat protein, animal fat, particle size

## Abstract

The current study aimed to investigate the effects of ultrasound-assisted emulsification on the emulsifying and rheological properties of myofibrillar protein (MP) pork fat emulsions under different protein/fat ratios. Changes in emulsion profile, confocal laser scanning microscope images, cryo-scanning microscope images, particle size, protein solubility, surface hydrophobicity and free sulfhydryl groups were determined. Ultrasound significantly increased the emulsifying activity, the emulsifying stability and the flow index for all emulsions, while it decreased the viscosity coefficient of emulsions except for the treatment of protein/fat ratio of 1:15. The results showed that sonication reduced the particle size of the fat particles and evenly distributed the emulsion droplets. Sonication moved the distribution curve of droplet size to the smaller particle size direction and decreased the D3,2 and D4,3 values of emulsion. Sonication resulted in increased bindings between protein hydrophobic groups and fat particles. After ultrasound treatment, more sulfhydryl groups were exposed to aqueous solution, which might decrease the protein solubility in aqueous solution. Ultrasound-assisted emulsification could directly enhance the emulsifying and rheological properties of MP-stabilized pork fat emulsions at different protein/fat ratios, in particular at the ratio of 1:10.

## 1. Introduction

The mixtures of myofibrillar protein (MP) and fat largely determine the texture, juiciness, flavor and appearance of emulsified meat products [1,2]. Recently, the high-energy emulsification methods such as ultrasound, high-pressure homogenization, high-speed homogenization and microfluidizer have been used to emulsify protein-stabilized emulsions [3]. Among the above emulsification methods, high-intensity ultrasound is gradually arousing the interest of researchers.

High-intensity ultrasound is a mechanical wave with the intensity ranging from 1 to 1000 W·cm^−2^ and the frequency being between 20 and 100 kHz [4]. As a green processing technology, ultrasound is used for many food processing methods such as emulsifying, tenderization, freezing, thawing, drying and extraction [5,6]. The main effect of ultrasound on the emulsion is attributed to acoustic cavitation [7]. The cavitation bubbles could suddenly collapse during the ultrasound wave transmission, leading to occurrence of the acoustic cavitation [4].

The applications of ultrasound to produce protein-stabilized emulsions have been widely reported, mostly focusing on preparing milk or plant protein stabilized vegetable oil emulsions [8]. For example, Qayum et al. [9] used ultrasound to emulsify α-lactalbumin and soybean oil emulsions and found that their emulsifying activity and emulsifying stability were significantly increased. As for emulsions prepared with soybean protein and palm oil, soybean oil, rapeseed oil and medium-chain triglyceride oil, ultrasound emulsification significantly increased the emulsifying stability and decreased the droplet size [10]. Ultrasound has also been used to prepare emulsions with whey protein isolate, whey protein concentrate, sodium caseinate, pea protein, rice protein and almond protein [11,12,13,14,15,16]. However, meat protein stabilized animal fat emulsions with the treatment of sonication have not reported, as far as we know.

The changes in the emulsifying properties of MP under different conditions have been widely studied. Fujiwara et al. [17] found that the conjugate of MP with dextran through the Maillard reaction had excellent emulsifying properties compared with MP (MP content of 3 g·L^−1^ and corn oil content of 25%). Li et al. [18] reported that the MP olive oil emulsions (with 10% oil) had a better stability within 48 h when the homogenization speed was at 4000 and 8000 rpm. Cha et al. [19] achieved a better emulsifying activity and emulsifying stability of MP-lecithin-soybean oil emulsions (with 10 g·L^−1^ MP and 10% soybean oil) after high-pressure homogenization treatment. However, the above studies about the emulsifying properties of MP mainly focused on relatively high fat conditions, which were not conducive to the development of low-fat meat products. This work aimed to study the influence of ultrasound as an emulsification method on the emulsifying properties of MP emulsions under different fat concentrations. Pork fat is solid at room temperature (25 °C), which may affect the emulsification effect of ultrasound. Therefore, we hypothesized that the influence of ultrasound-assisted emulsification on the different fat concentration MP emulsions might be different. The findings in this article could provide some guidance for ultrasound applications in the processing of emulsified meat products and the preparation of emulsions with different fat concentrations.

## 2. Materials and Methods

### 2.1. Materials

Six porcine longissimus dorsi muscles (24 h post-mortem) and pork fat were randomly sampled at Sushi Meat Co., Ltd. (Huai’an, China), then frozen at −20 °C and were used within one week. Bovine serum albumin (BSA) and other chemicals were purchased from Sinopharm Chemical Reagent Co., Ltd. (Shanghai, China), and all chemicals were analytical grade.

### 2.2. Extraction of MP and Pork Fat

The extraction and the purification of pork myofibrillar protein (MP) were according to the method of Park et al. [20] with some modifications. Briefly, pork was thawed at 4 °C for 8 h and then cut into small pieces (around 0.5 × 0.5 × 0.5 cm^3^). The sample was then homogenized three times (10 s for each time) at 10,000 rpm using 4 volumes of buffer I (0.1 mol·L^−1^ KCl, 10 mmol·L^−1^ K_2_HPO_4_, 2 mmol·L^−1^ MgCl_2_, 1 mmol·L^−1^ EGTA and 0.5 mmol·L^−1^ dithiothreitol, pH 7.0, 4 °C). After centrifuging the homogenates at 4 °C and 2000× *g* for 20 min (Avanti J-26XP, Beckman Coulter, Brea, CA, USA), the sediments were collected, and the above procedure was repeated twice using buffer I. After that, the sediments were resuspended in 4 volumes of buffer II (0.1 mol·L^−1^ KCl, 1 mmol·L^−1^ NaN_3_, pH 6.0, 4 °C), and the homogenates were filtered with a single-layer gauze (Medical Equipment Factory of Shanghai Medical Instruments Co., Ltd., Shanghai, China). After centrifuging the homogenates at 2000× *g* and 4 °C for 20 min, the sediments were collected, and the above procedures were repeated twice using buffer II. The sediments after six rounds of centrifugation were the extracted proteins. The protein concentrations were determined by the Lowry method (Folin-phenol reagent method) with BSA as standard and used within 48 h.

After washing and drying pork fat, the extraction and the purification of fat were performed as the method described by Zhou et al. [21]. The extracted fat was placed at −20 °C and used within 3 months. The fat was placed at 45 °C for 30 min before usage.

### 2.3. Ultrasound-Assisted Emulsification

The appropriate amount of fat was mixed with MP under phosphate buffer (0.6 mol·L^−1^ KCl, 10 mmol·L^−1^ KH_2_PO_4_, pH 6.0, 4 °C). The protein concentration was 30 g·L^−1^ and fat contents were 2, 3, 6, 30, 150, 300 and 450 mL·L^−1^ according to our previous studies [22]. The ratios of protein to fat were 15:1, 10:1, 5:1, 1:1, 1:5, 1:10 and 1:15 (*w*/*v*), and the total volumes of all samples were 10 mL. After mixing the sample evenly with a homogenizer (T10 basic ULTRA-TURRAX, IKA, Staufen, Germany) at 9500 rpm for 60 s, ultrasound emulsification treatment was carried out at the output power of 240 W for 6 min with pulse durations of 1 s on and 3 s off with a 20 kHz ultrasound processor (650 W full power, JY98-IIIN, Scientz Biotechnology Co., Ltd., Ningbo, China) equipped with a 6 mm (diameter) titanium probe [23]. The ultrasound intensity was determined by the method described by Jambrak et al. [24], and the ultrasound intensity was 12.38 W·cm^−2^. During sonication, samples were placed in an ice-water bath, and the sample temperature was maintained below 20 °C. In this study, untreated samples (non-ultrasound) were used as controls and recorded as N1, N2, N3, N4, N5, N6 and N7 according to the order of change of MP and fat ratios. The sonicated samples were recorded as U1, U2, U3, U4, U5, U6 and U7. All samples were diluted to a protein concentration of 1 g·L^−1^ with phosphate buffer. The 30 g·L^−1^ protein concentration sample was used for the measurements of emulsifying properties, rheological properties, morphological structure and particle size. The 1 g·L^−1^ protein concentration sample was used for the measurements of protein solubility, surface hydrophobicity (S_0_-ANS) and free sulfhydryl (SH) group content.

### 2.4. Emulsifying Properties

The determination of emulsifying activity of MP was followed with the method as described by Pearce and Kinsella [25] with minor modifications. Briefly, the fresh sample and the sample after 10 min of 20 μL aliquots were dispersed into 20 mL of 0.1% sodium dodecyl sulfate solution. The absorbance of solutions was measured at a wavelength of 500 nm. The emulsifying activity index (EAI) and the emulsifying stability index (ESI) were calculated following Equations (1) and (2):

(1) $${\mathrm{EAI}\;(m}^{2}/g)=\frac{2\;\times \;2.303\;\times \;1000}{C\;\times \;\left(1-\varphi \right){\;\times \;10}^{4}}{\;\times \;A}_{0}$$

(2) $$\mathrm{ESI}\;\left(\%\right)=10\;\times \;\frac{A_{0}}{A_{0}-A_{10}}$$

where C represents the protein concentration (g·mL^−1^) before emulsification, φ is the oil volume fraction (*v*/*v*) of samples, A_0_ and A_10_ represent the absorbance at time zero and after 10 min, and 1000 is the dilution factor.

### 2.5. Rheological Properties

The viscosity coefficients (k), the flow index (n) and the viscosity of MP-pork fat emulsions were carried out using a rotational rheometer (MCR302, Anton Paar, Graz, Austria) equipped with 50 mm parallel plate. The parameters were monitored as follows: gap, 0.5 mm; shear rate, 1 to 1000 s^−1^. Power law Equation (3) was used to calculate the k and the n values:

(3) $${\tau =k\;\times \;\delta }^{n_{1}}$$

where τ is the shear stress (Pa), k is the viscosity coefficients (Pa·s^n^), δ is the shear rate (s^−1^) and n_1_ is the flow index obtained from power law equation. However, the value of k depends on the n values, and it is not conducive to compare the k values of samples with different n values. To avoid this situation, a modified power law Equation (4) according to Trujillo-Cayado et al. [26] was applied:

(4) $$\tau =\tau_{1}\times {\left(\frac{\delta }{1s^{-1}}\right)}^{n_{2}}$$

where τ_1_ is the shear stress value at 1 s^−1^, and n^2^ is the flow index derived from modified power law equation. The value of viscosity was obtained when the shear rate was 100 s^−1^.

### 2.6. Morphological Structure

#### 2.6.1. Emulsion Observation

To observe the distribution of emulsion, an optical microscope (EX 30, Sunny Optical Technology Co., Ltd., Ningbo, China) was used under a 10× magnifier.

#### 2.6.2. Emulsion Droplet Observation

After staining the emulsions (1 mL) with 80 μL 0.1% (*m*/*v*) Nile Blue and 0.1% (*m*/*v*) Nile Red mixture for 8 h in darkness, the oil droplets before and after ultrasound emulsification were observed using a confocal laser scanning microscope (CLSM, TCS SP8 X, Leica, Wetzlar, Germany). The excitation wavelengths were 488 nm and 633 nm, respectively. The green color represents oil, and red color in this test represents protein in this work.

#### 2.6.3. Emulsion Ultrastructure Observation

The ultrastructure of emulsions was analyzed using a cryo-scanning electron microscopy (cryo-SEM, SU 3500, Hitachi Corporation, Tokyo, Japan) at 1000× under a 5 kV accelerating voltage. First, conductive carbon glue was applied on the sample stage, and the samples were stuck on the conductive carbon glue. After that, the samples were put into the liquid nitrogen slush for 30 s, and then they were transferred to the sample preparation chamber for sublimation gold plating using the low-temperature freezing preparation transfer system under vacuum. The sample was sublimated at −90 °C for 10 min and then sputtered with gold for 60 s at a current of 10 mA.

### 2.7. Droplet Size

Malvern 3000 dynamic laser particle size analyzer (Malvern Instruments Ltd., Malvern, Nottinghamshire, UK) was used to determine the droplet size. Refractive index and adsorption of emulsion particle were 1.436 and 0.001, and the refractive index of phosphate buffer was 1.330. The D10, D50, D90, D3,2 and D4,3 values were obtained from this test. The Span value was calculated by following Equation (5):

(5) $$\mathrm{Span}=\frac{D_{90}-D_{10}}{D_{50}}$$

### 2.8. Protein Solubility

The MP solubility in emulsions was measured by the method as described by Anon et al. [27] with slight modifications. After centrifuging the aliquots at 10,000× *g* for 20 min, the aqueous solution was taken out with a pipette to determine the protein concentration by Lowry method with BSA as standard. Protein solubility (%) was expressed as the ratio of protein concentration in aqueous solution before and after centrifugation.

### 2.9. Surface Hydrophobicity (S_0_-ANS)

To determine the S_0_-ANS of emulsions, 8-anilino-1-naphthalenesulphonic acid (ANS) was used as a fluorescence probe, and the method was performed as described by Zhang et al. [28]. Briefly, 10 μL aliquots of ANS solutions (8 mmol·L^−1^ ANS, 0.1 mol·L^−1^ KH_2_PO_4_, pH 6.0) were added into 2 mL emulsions and thoroughly mixed with a vortex. After the mixture was put in the dark at 25 °C for 20 min, the measurement of fluorescence intensity was carried out with a fluorescence spectrophotometer (F-7000, Hitachi Corporation, Tokyo, Japan) at an excitation wavelength of 374 nm and an emission wavelength of 485 nm.

### 2.10. Free Sulfhydryl (SH)

The free SH group content of emulsions was determined by Ellman’s reagent as the method described by Liu et al. [29] with minor modifications. After mixing 2 mL emulsions and 50 μL Ellman’s reagent (10 mol·L^−1^, 5,5-dithiobis [2-nitrobenzoic acid], 10 mol·L^−1^ K_2_HPO_4_, pH 6.0), the mixtures were kept at 4 °C for 60 min. The absorbance of mixtures was measured at 412 nm, and the free SH group content was calculated following Equation (6):

(6) $$\mu \mathrm{mol}\;\mathrm{SH}/g\;\mathrm{protein}={73.53\;\times \;A}_{412}\;\times \;{1.025\;\times \;C}^{-1}$$

The A412 represents the absorbance of mixtures, 1.025 represents the dilution factor, and C represents the concentration of MP in emulsions.

### 2.11. Statistical Analysis

SPSS software (Ver. 24, IBM Corporation, Armonk, NY, USA) was used for data analysis. All values were presented as means ± standard error. The one-way analysis of variance was used to determine the significance, and Duncan multiple tests at *p* ≤ 0.05 level indicated a significant difference.

## 3. Results and Discussion

### 3.1. Emulsifying Properties

**Figure 1:**
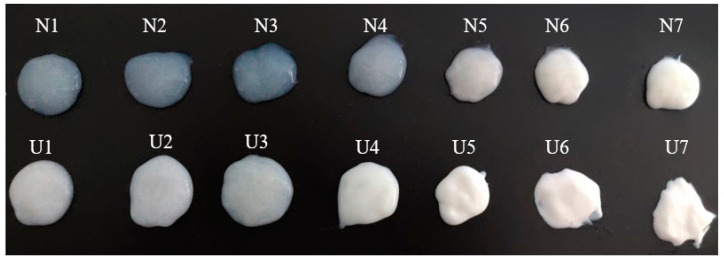
The profiles of emulsions (30 g·L^−1^) with non-ultrasound and ultrasound emulsification treatment.

Figure 1 shows the profile of emulsions with non-ultrasound and ultrasound treatment. It was apparent that the ultrasonic treatment provided the samples with a more pronounced emulsion-white appearance. Table 1 shows the changes in the EAI and ESI of MP-pork fat emulsions (under different fat conditions) with non-ultrasound and ultrasound treatment. With the increase in fat volumes, the EAI of emulsions significantly increased for both non-ultrasound and ultrasound groups. The value of EAI significantly increased after sonication, especially for U6 groups. The ESI gradually decreased as the fat volume increased for non-ultrasound-treated groups, while it gradually decreased and then increased for the ultrasound-treated groups. The ESI of emulsions also significantly increased after ultrasound treatment.

As an amphiphilic macromolecule, MP can be used as an effective emulsifier in meat products to reduce oil–water interfacial tension [30,31]. Unlike emulsifiers with low molecular weights that diffuse rapidly to the interface to give strong emulsion-forming properties, MP diffuses slowly to the interface; thus, it can not easily form a strong emulsion [3]. This might be a reason for the lower EAI and ESI values of the non-ultrasound samples. The cavitation of ultrasound reduced the particle size of fat droplets [32] and MP [28], resulting in an increase in the contact level between MP and fat particles (Figure S1 and Figure 3). Thereby, the EAI and the ESI of the emulsions were both significantly increased after ultrasound treatment. As the fat content increased, more protein acted as an emulsifier and participated in the formation of the emulsion, thereby increasing its EAI value. When the ratio of protein to fat decreased from 1:10 to 1:15, the EAI and ESI values of sonicated emulsions decreased, which might be due to the difficulty of further decreasing fat particle size under the current ultrasound intensity (Figure 3).

### 3.2. Rheological Properties

**Figure 2:**
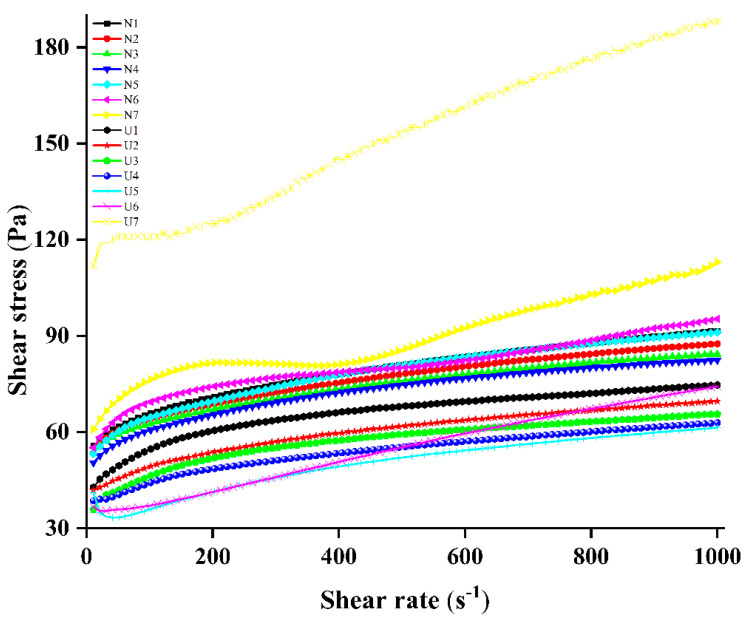
Changes in the shear rate and shear stress of emulsions as affected by high-intensity ultrasound emulsification.

The static rheological behavior is used to describe changes in fluid properties under external forces. Figure 2 represents the effect of ultrasound treatment on the flow curves of MP pork fat emulsions. Table 1 shows that the changes of rheological properties of emulsions in higher shear rate range were fitted by a power law equation. All emulsions exhibited a pseudoplastic behavior (n < 1, both for n_1_ and n_2_). Sonication significantly increased the n_1_ values of all emulsions and increased the n_2_ values except for the sample with a protein-to-fat ratio at 1:15. The viscosity values were decreased after ultrasound treatment when the protein-to-fat ratio was changed from 15:1 to 1:10, and then increased at the ratio of 1:15 after sonication. Ultrasound treatment firstly caused a significant increase (N1 to N6 and U1 to U6) and then a significant decrease in the k values (N7 and U7). The amount of fat content also significantly affected the k and n_1_ values of emulsions, especially after sonication. A significant decrease in k values and an increase in n values for the ultrasound-treated emulsions were shown when the fat content increased (U1 to U6), while an increase in k and n_2_ values and a decrease in n_1_ values were detected when the fat content further increased (U7).

During sonication, protein molecules and fat particles move rapidly due to the cavitation and microstreaming. These changes could unfold protein chains [24], decrease the size of fat particles [32], enhance the emulsification effect of solutions (increased EAI showed in Table 1) and lead to the increase in n values and the decrease in k values and viscosity (U1 to U6). Wang et al. [23] and Zhang et al. [28] used high-intensity ultrasound to treat MP solutions and also found ultrasound treatment decreased k values and increased n values of MP solutions. Similar results were also reported in soybean protein by Hu et al. [33]. The different trends of the U7 sample might be because the emulsions mainly exhibited the fat fluid properties (different curves and lower R^2^ values) under high-fat conditions.

### 3.3. Optical Microscope

Figure S1 shows the droplet distribution of emulsions observed from optical microscope with non-ultrasound and ultrasound treatment. The fat particles of non-ultrasound samples were unevenly dispersed in the MP solution and aggregated into large fat globules as the fat content increased. After sonication, the size of fat particles decreased and evenly dispersed in the MP solution. For samples from N1 to N4 and from U1 to U4, ultrasonic treatment also decreased the size of emulsion bubbles, which even disappeared completely at the treatments of N4 and U4. These changes are also observed in Figure 1. These results indicate that ultrasound treatment promoted the formation and uniform distribution of small fat droplets of all samples, which increased the emulsifying properties of samples. Li et al. [34] also found that the formation of smaller fat droplets contributed to better emulsifying properties when ultrasound was used to treat MP and then to form emulsions.

### 3.4. CLSM and Cryo-SEM

**Figure 3:**
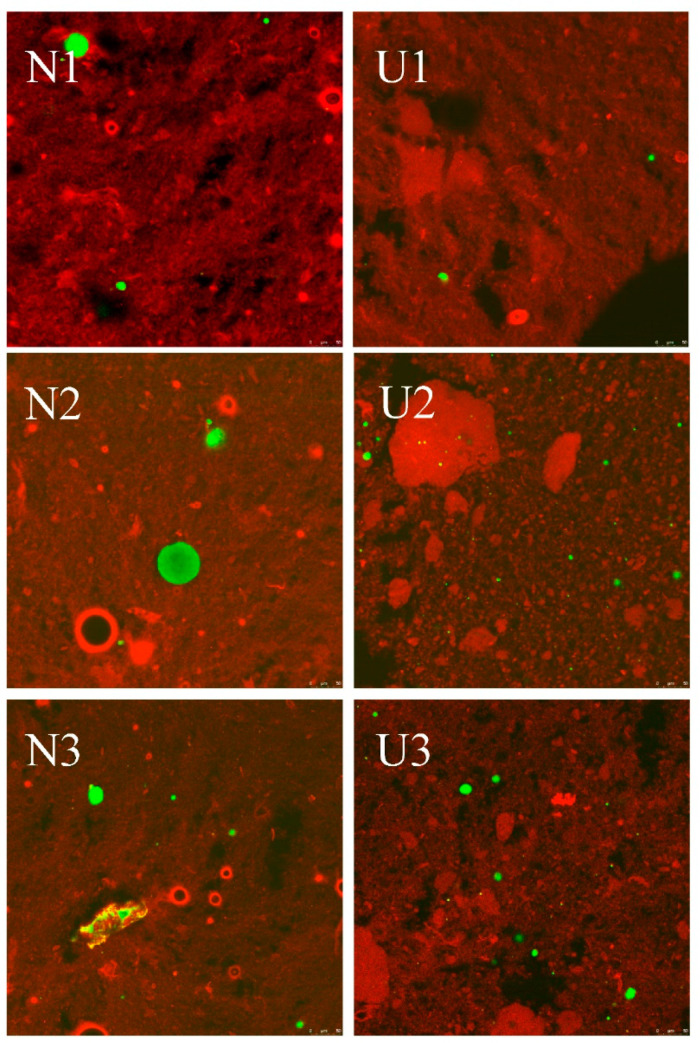

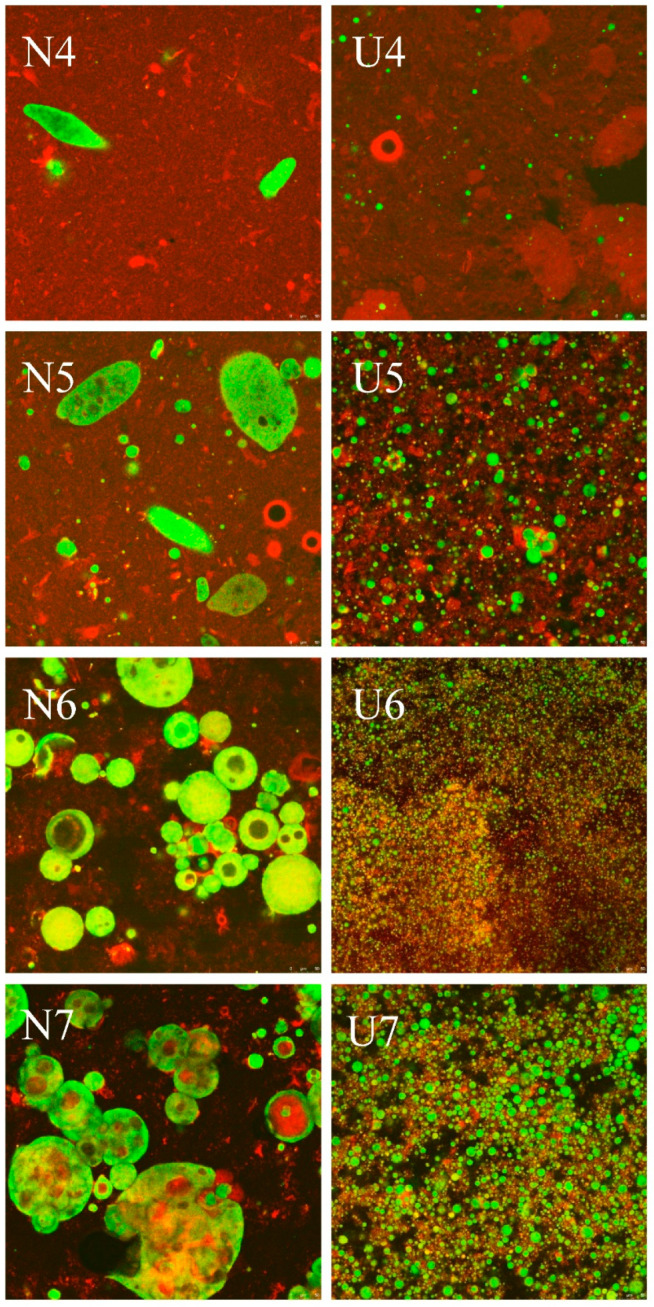
The CLSM images of myofibrillar protein pork fat emulsions as affected by high-intensity ultrasound emulsification. The red color represents MP, and green color represents pork fat in this figure.

**Figure 4:**
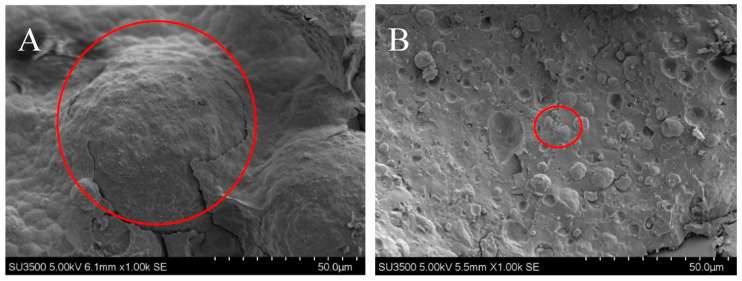
The Cryo-SEM images of the 1:10 emulsions before (**A**) and after (**B**) high-intensity ultrasound emulsification. The spherical objects in the red circle represent emulsion droplets.

CLSM has a wide range of applications in reflecting the properties of the food emulsions [35]. The CLSM images of emulsions affected by fat concentrations and sonication are shown in Figure 3. For no-ultrasound treated samples, the oil droplet size gradually increased as the oil concentration increased, especially for the samples with the protein-to-oil ratio being lower than 1:5. The above changes might be attributed to insufficient emulsification, which caused the emulsion droplets to aggregate during the staining process. Sonication treatment significantly changed this phenomenon, especially for the sample with a protein-to-fat ratio of 1:10. However, the influences of ultrasound emulsification on the emulsion were different as the ratio of protein to fat changed. Most proteins did not participate in the formation of protein-coated emulsions when the protein-to-oil ratio was higher than 1:10, while most proteins participated in the formation of emulsions and formed small emulsion droplets when the protein-to-fat ratio was at 1:10. However, the oil droplet size increased obviously when the ratio was at 1:15. The increased oil droplet size for sample with the ratio 1:15 might be due to the decreased temperature causing the fat to change from liquid to solid, which affected the overall properties of the emulsion. Therefore, the oil droplets did not decrease to a smaller size under the present ultrasound intensity. The most obvious effect of ultrasound emulsification on emulsions was at the 1:10 ratio, and thus cryo-SEM was used to further explore the changes of protein-coated oil droplets at this ratio. The influences of sonication on the emulsions are shown in Figure 4. The size of protein-coated oil droplets was significantly decreased by sonication. This change further confirms that sonication could decrease the emulsion droplet size to form smaller protein-coated oil droplets.

### 3.5. Particle Size

**Figure 5:**
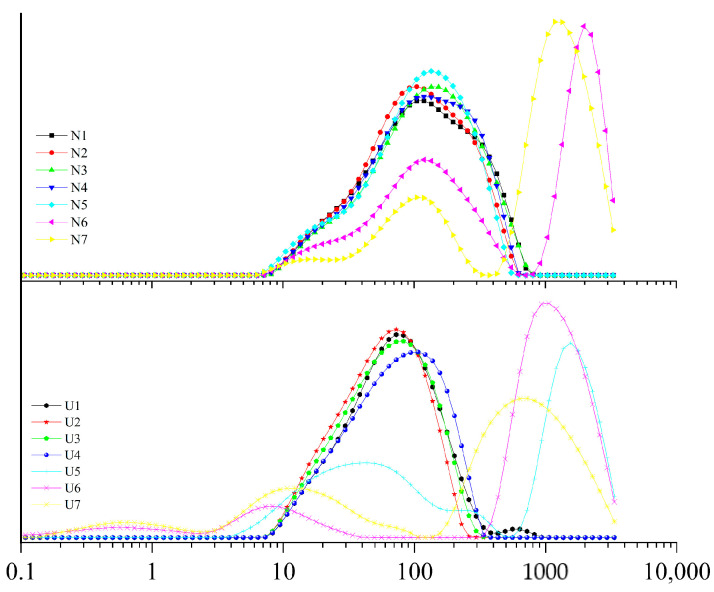
The droplet distribution of emulsions as affected by ultrasound emulsification.

Table 2 shows the changes of Span, D3,2 and D4,3 of emulsions as affected by ultrasound. Sonication decreased the values of D3,2 and D4,3 of all ratio emulsions, especially for the ratio at 1:10, for which the D3,2 value decreased from 130.2 to 1.7 μm. The D4,3 value of non-ultrasound treated sample had no significant change firstly as the ratio decreased from 15:1 to 1:5. Then, the D4,3 values significantly increased when the ratio reached 1:10, and no significant change was observed when the ratio decreased to 1:15. The D3,2 value firstly showed no significant change when the ratio decreased from 15:1 to 1:10 and significantly increased when the ratio attained 1:15. However, the influence of the protein-to-fat ratio on sonicated emulsion was different. As the oil concentration increased, the D3,2 value of sonicated emulsions firstly decreased and then increased, and it reached a minimum at the ratio of 1:10. The value of D4,3 firstly showed no significant change as the ratio was changed from 15:1 to 1:10, and it significantly increased when the ratio reached 1:15. The influence of ultrasound emulsification on the span value firstly had no significant change when the ratio decreased from 15:1 to 1:5, and then it significantly increased when the ratio was at 1:10 and 1:15. Figure 5 shows the changes of droplet size distribution as affected by ultrasound emulsification. Sonication moved the distribution curve of droplet size to the left (smaller particle size direction), especially for the U6 and U7 samples, which showed smaller droplets after sonication.

The decreased droplet size by sonication indicated that sonication caused large particles of the emulsion droplets to be dispersed into small particles by the high shear forces and cavitation. The value of D3,2 is more sensitive to smaller particles, while the D4,3 value is more sensitive to large particles. Due to the heterogeneity of MP pork fat emulsion, the D4,3 value was higher than D3,2 value for all emulsions. The droplet size of an emulsion is a critical parameter for most emulsion characteristics. A small emulsion particle size and a uniform emulsion distribution generally indicate that the emulsion has good emulsifying property and stability [30,36]. Zhao et al. [37] also found that a smaller particle size and homogeneous dispersion of fat particles contributed to the stabilization of the isolated chicken protein–soybean oil emulsion. The non-sonicated sample had many large particles, which made the emulsion prone to flocculation and was not conducive to the stability of the emulsion [38]. Therefore, the EAI and the ESI values were increased after ultrasound emulsification (Table 1). Compared with the emulsions with protein-to-fat ratios ranging from 15:1 to 1:1, the effects of sonication on the emulsion droplet size were more significant when the ratio ranged from 1:5 to 1:15, especially for the ratio at 1:10. The significantly increased span values of the 10:1 and 15:1 samples might be due to the wide range of droplet size distribution (Figure 5).

### 3.6. Protein Solubility

The protein solubility could affect or contribute to the functional properties of most proteins. After ultrasound emulsification, the MP solubility in emulsions significantly decreased (Table 3); in particular the MP solubility of U6 decreased from 46.54% to 10.95%. This result is contrary to the results of many researchers who found that ultrasonic treatment increased the solubility of proteins [23,28,33,39]. The inconsistent results are most likely because they used ultrasound to directly treat proteins, while we used ultrasound as an emulsification method to treat proteins and fat mixtures. When ultrasound was used to treat mixtures, even if cavitation disrupted the intermolecular association of protein aggregates and unfolded both the hydrophilic and hydrophobic groups [39], more hydrophobic groups were combined with fat particles by ultrasound emulsification. The greater amalgamation between hydrophobic amino acid side chains and fat particles resulted in a higher binding force than that of the protein molecules and the aqueous solution. This might be an important reason for the decreased protein concentration in the aqueous solution and the gradually decreased solubility of the sonicated sample (U1 to U6) as the fat content increased.

### 3.7. S_0_-ANS

Protein S_0_-ANS affects its binding ability to fat particles, and higher S_0_-ANS means that proteins have good emulsifying properties [36,40]. Sonication significantly increased the S_0_-ANS of MP in emulsions under all fat conditions (Table 3). The values of S_0_-ANS significantly increased within lower fat additions (N1 to N4 and U1 to U3), and then significantly decreased with the increase in fat content (N4 to N7 and U3 to U7). The increase in S_0_-ANS after sonication might be due to the cavitation phenomenon, which causes the protein molecules to unfold and exposes the hydrophobic groups. The results are consistent with previous studies which showed that ultrasound treatment increased the S_0_-ANS of MP [23,28]. At lower fat levels (N1 to N4), the increased S_0_-ANS of the MP by fat might be due to the hydrophobic long chain of the fat, which facilitated the exposure of the protein hydrophobic groups [21]. As the fat content was further increased, the emulsification was enhanced, and more hydrophobic groups were combined with the fat particles, thereby decreasing the S_0_-ANS. The change in S_0_-ANS confirms that the hydrophobic amino acid side chains were combined with the fat particle after ultrasound emulsification, thereby reducing the protein solubility in aqueous solution (Table 3).

### 3.8. Free SH Groups

The free SH group content gradually increased as the fat content increased both for the non-ultrasound and ultrasound treated samples (Table 3). After sonication, the free SH group content increased under all fat conditions, suggesting that interior SH groups were exposed to the surface. Due to the different hydrophobicity effects of the side chains of each amino acid on the protein molecules, amino acid side chains with stronger hydrophobic properties (such as leucine, phenylalanine, valine, tryptophan, isoleucine and tyrosine) easily combined with fat, and the more hydrophobic amino acids (such as cysteine) were excluded [41]. Therefore, more SH groups were detected as the fat content increased.

### 3.9. Schematic Model

**Figure 6:**
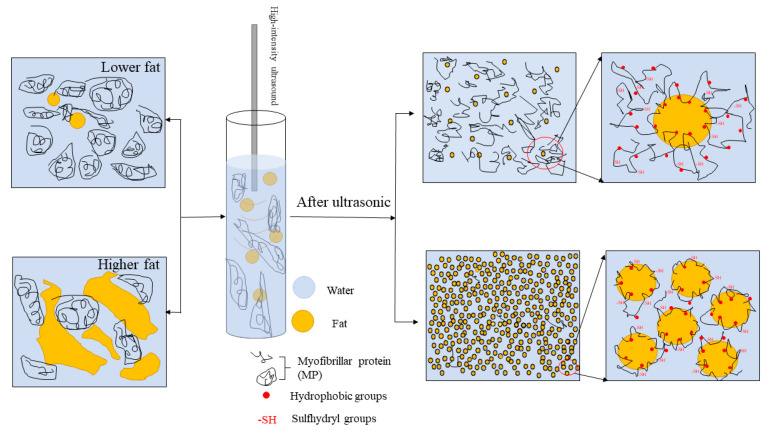
Schematic diagram illustrating the improvement of MP pork fat emulsion properties with high-intensity ultrasound emulsification.

Based on the above results, a schematic model as shown in Figure 6 is proposed to illustrate the influence of protein and fat ratio on the MP pork fat emulsions under high-intensity ultrasound emulsification. Sonication decreased the size of fat particles and exposed MP molecules for all emulsions. For lower fat emulsions (U1, U2, U3 and U4), almost all fats were emulsified by MP, while most MP molecules were spread in solutions. For higher fat emulsions (U5, U6 and U7), more MP molecules participated in the formation of protein-coated fat droplets as the fat concentration increased, and smaller emulsion droplets formed, especially for the protein-to-fat ratio of 1:10. After ultrasound emulsification was applied to prepare MP pork fat emulsions, the hydrophobic groups of MP tended to bind to fat particles, while SH groups were more likely to be exposed to the aqueous solution (Table 3).

## 4. Conclusions

Ultrasound-assisted emulsification could enhance the emulsifying activity index and emulsifying stability index of MP under different pork fat conditions, especially under higher fat conditions (the ratio of protein to fat at 1:10 and 1:15). Sonication reduced the flow index (n_1_) values of all emulsions and decreased the n_2_ values, except for the 1:15 sample. Ultrasonic treatment decreased the viscosity coefficient (k) value of the emulsions as the protein-to-fat ratio changed from 15:1 to 1:10, while it increased the k values when the protein and fat ratio was at 1:15. Ultrasonic treatment affected the emulsifying and rheological properties of the emulsion by reducing the diameter of the emulsion particles, and it allowed more protein hydrophobic groups to be combined with fat particles and exposed the sulfhydryl groups to aqueous solution. Compared with the MP emulsions of lower pork fat ratios (ratio during 1:15 to 1:1), ultrasound emulsification had better influence on emulsions with higher pork fat ratios (ratio during 1:5 to 1:15), especially at the ratio of 1:10. Based on the results of the current study, ultrasound is an efficient method for preparing MP-stabilized pork fat emulsions, but future research should further compare the effects of ultrasound on MP emulsions with other emulsification methods.

## Figures and Tables

**Table 1:** Changes in emulsifying activity index (EAI), emulsifying stability index (ESI), viscosity coefficients (k), flow index (n_1_ and n_2_), coefficient of determination (R^2^) and viscosity (when the shear rate was 100 s^−1^) of emulsion before (non-ultrasound, N) and after ultrasound (U) emulsification treatment.

Parameters		15:1	10:1	5:1	1:1	1:5	1:10	1:15
EAI (m^2^/g)	N	0.02 ± 0.01 ^eB^	0.06 ± 0.01 ^eB^	0.09 ± 0.02 ^deB^	0.18 ± 0.04 ^dB^	0.32 ± 0.04 ^cB^	0.74 ± 0.08 ^bB^	1.20 ± 0.13 ^aB^
	U	0.09 ± 0.01 ^dA^	0.18 ± 0.01 ^dA^	0.53 ± 0.02 ^cdA^	2.14 ± 0.11 ^cA^	6.09 ± 0.20 ^bA^	27.36 ± 1.13 ^aA^	25.65 ± 3.09 ^aA^
ESI (%)	N	25.46 ± 5.03 ^aB^	22.48 ± 2.44 ^abB^	21.89 ± 3.86 ^abB^	20.41 ± 9.00 ^abB^	19.90 ± 1.47 ^abB^	15.68 ± 1.09 ^bB^	17.52 ± 1.14 ^bB^
	U	65.35 ± 10.21 ^aA^	32.06 ± 4.32 ^cdA^	28.49 ± 2.19 ^dA^	42.80 ± 10.67 ^bcA^	47.07 ± 8.52 ^bA^	45.68 ± 7.89 ^bA^	30.98 ± 5.08 ^dA^
k (Pa·s^n^)	N	37.1 ± 0.7 ^bA^	36.8 ± 0.6 ^bA^	37.3 ± 0.6 ^bA^	34.8 ± 0.5 ^cA^	34.8 ± 0.6 ^cA^	39.8 ± 0.9 ^aA^	37.3 ± 1.8 ^bB^
	U	30.0 ± 0.1 ^bB^	25.6 ± 0.3 ^cB^	24.1 ± 0.1 ^cdB^	23.1 ± 0.3 ^dB^	14.6 ± 0.6 ^eB^	9.3 ± 0.6 ^fB^	52.0 ± 2.5 ^aA^
n_1_	N	0.127 ± 0.003 ^cA^	0.122 ± 0.003 ^cdB^	0.115 ± 0.003 ^dB^	0.123 ± 0.002 ^cdB^	0.137 ± 0.003 ^bB^	0.118 ± 0.004 ^dB^	0.146 ± 0.008 ^aB^
	U	0.132 ± 0.001 ^eA^	0.142 ± 0.002 ^deA^	0.144 ± 0.001 ^dA^	0.142 ± 0.002 ^deA^	0.205 ± 0.006 ^bA^	0.292 ± 0.001 ^aA^	0.179 ± 0.008 ^cA^
R^2^	N	0.956	0.964	0.961	0.976	0.966	0.934	0.818
	U	0.998	0.985	0.998	0.979	0.930	0.915	0.863
n_2_	N	0.077	0.082	0.076	0.085	0.087	0.086	0.089
	U	0.094	0.093	0.101	0.098	0.164	0.123	0.070
Viscosity (Pa·s)	N	0.645	0.629	0.615	0.595	0.634	0.681	0.750
	U	0.537	0.469	0.455	0.434	0.353	0.364	1.19

Different lowercase letters (a–f) in the same line and different uppercase letters (A and B) in the same column indicate significant difference (*p* < 0.05) among samples.

**Table 2:** Changes in D3,2, D4,3 and span value of emulsions before (non-ultrasound, N) and after ultrasound (U) emulsification treatment.

Parameters		15:1	10:1	5:1	1:1	1:5	1:10	1:15
D3,2 (μm)	N	67.9 ± 4.7 ^bA^	66.7 ± 2.5 ^bA^	67.4 ± 2.2 ^bA^	65.3 ± 4.3 ^bA^	64.1 ± 3.6 ^bA^	130.2 ± 16.9 ^bA^	410.5 ± 162.2 ^aA^
	U	50.7 ± 3.2 ^aB^	42.7 ± 1.0 ^bB^	44.4 ± 1.4 ^bB^	44.5 ± 1.6 ^bB^	26.1 ± 1.8 ^cB^	1.7 ± 1.2 ^eB^	5.0 ± 2.3 ^dB^
D4,3 (μm)	N	157.8 ± 19.7 ^bA^	153.2 ± 12.4 ^bA^	146.7 ± 8.6 ^bA^	144.3 ± 16.8 ^bA^	153.2 ± 16.9 ^bA^	983.8 ± 96.7 ^aA^	1067.7 ± 340.2 ^aA^
	U	97.1 ± 12.0 ^bB^	88.5 ± 13.4 ^bB^	89.8 ± 12.2 ^bB^	81.4 ± 13.1 ^bB^	133.7 ± 83.9 ^bA^	131.8 ± 58.3 ^bB^	544.0 ± 222.0 ^aB^
Span	N	2.8 ± 0.2 ^abA^	2.9 ± 0.3 ^aA^	2.6 ± 0.2 ^abA^	2.6 ± 0.1 ^abA^	2.7 ± 0.6 ^aA^	2.5 ± 0.4 ^abB^	1.7 ± 0.2 ^aB^
	U	2.4 ± 0.2 ^bA^	2.9 ± 0.7 ^bA^	2.7 ± 0.5 ^bA^	2.2 ± 0.3 ^bA^	8.6 ± 6.2 ^bA^	38.9 ± 14.9 ^aA^	2.5 ± 0.5 ^bA^

Different lowercase letters (a–e) in the same line and different uppercase letters (A and B) in the same column indicate significant difference (*p* < 0.05) among samples.

**Table 3:** Changes in protein solubility, surface hydrophobicity (S_0_-ANS) and free sulfhydryl (SH) group content of emulsions before (non-ultrasound, U) and after ultrasound (U) emulsification treatment.

Parameters		15:1	10:1	5:1	1:1	1:5	1:10	1:15
Solubility (%)	N	42.97 ± 1.68 ^bA^	42.85 ± 0.63 ^bA^	44.11 ± 2.58 ^bA^	45.26 ± 2.23 ^bA^	51.09 ± 0.85 ^aA^	46.54 ± 3.15 ^bA^	36.96 ± 0.59 ^cA^
	U	32.45 ± 0.31 ^aB^	28.55 ± 0.20 ^bB^	28.59 ± 0.82 ^bB^	23.74 ± 1.06 ^cB^	16.87 ± 0.73 ^dB^	10.95 ± 0.30 ^eB^	18.03 ± 1.43 ^dB^
S_0_-ANS	N	358 ± 9 ^cB^	374 ± 11 ^bcB^	389 ± 14 ^bB^	430 ± 18 ^aB^	316 ± 16 ^dB^	323 ± 15 ^dB^	324 ± 27 ^dA^
	U	607 ± 16 ^bA^	622 ± 6 ^bA^	687 ± 24 ^aA^	536 ± 68 ^cA^	409 ± 19 ^dA^	434 ± 19 ^dA^	373 ± 59 ^dA^
Free SH (μmol/g)	N	35 ± 6 ^bA^	39 ± 9 ^abB^	45 ± 8 ^abB^	45 ± 4 ^abB^	50 ± 11 ^aB^	49 ± 7 ^aB^	48 ± 4 ^aB^
	U	42 ± 2 ^eA^	52 ± 6 ^deA^	59 ± 7 ^dA^	74 ± 6 ^cA^	138 ± 8 ^bA^	152 ± 9 ^aA^	156 ± 12 ^aA^

Different lowercase letters (a–e) in the same line and different uppercase letters (A and B) in the same column indicate significant difference (*p* < 0.05) among samples.

## Data Availability

No data.

## Supplementary Materials

The following are available online at https://www.mdpi.com/article/10.3390/foods10061201/s1, Figure S1: The distribution of oil droplets in emulsions as affected by high-intensity ultrasound emulsification.
