# Supplementary material for: Effects of Ultrasound-Assisted Emulsification on the Emulsifying and Rheological Properties of Myofibrillar Protein Stabilized Pork Fat Emulsions
Source: Foods. 2021 May 26;10(6):1201. doi: 10.3390/foods10061201 (PMC8226962; doi:10.3390/foods10061201)
Supplement: Supplementary file 1 [file foods-10-01201-s001.zip › foods-1184000-supplementary.pdf]

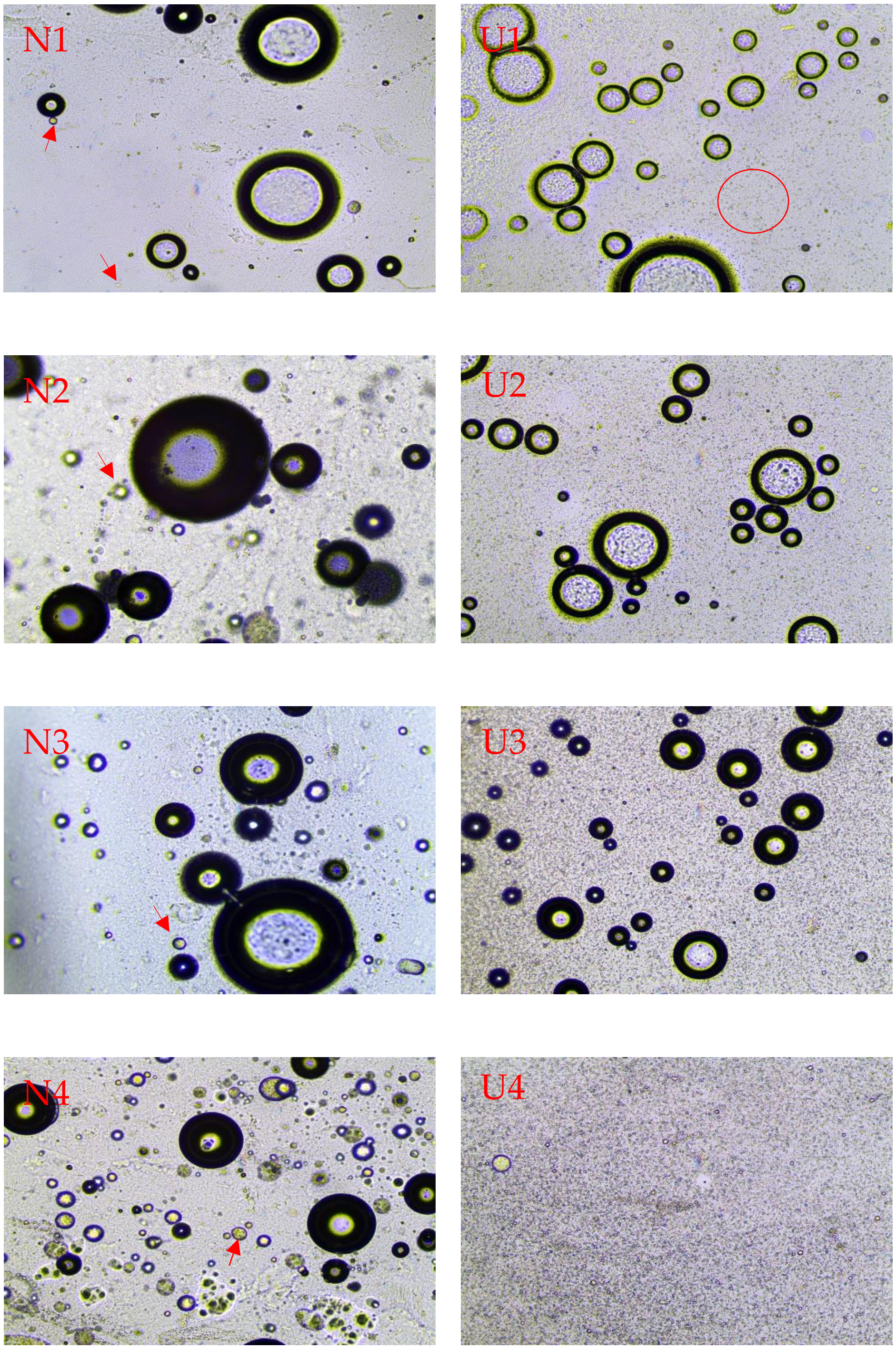
N1
U1
N2
U2
N3
U3
N4
U4

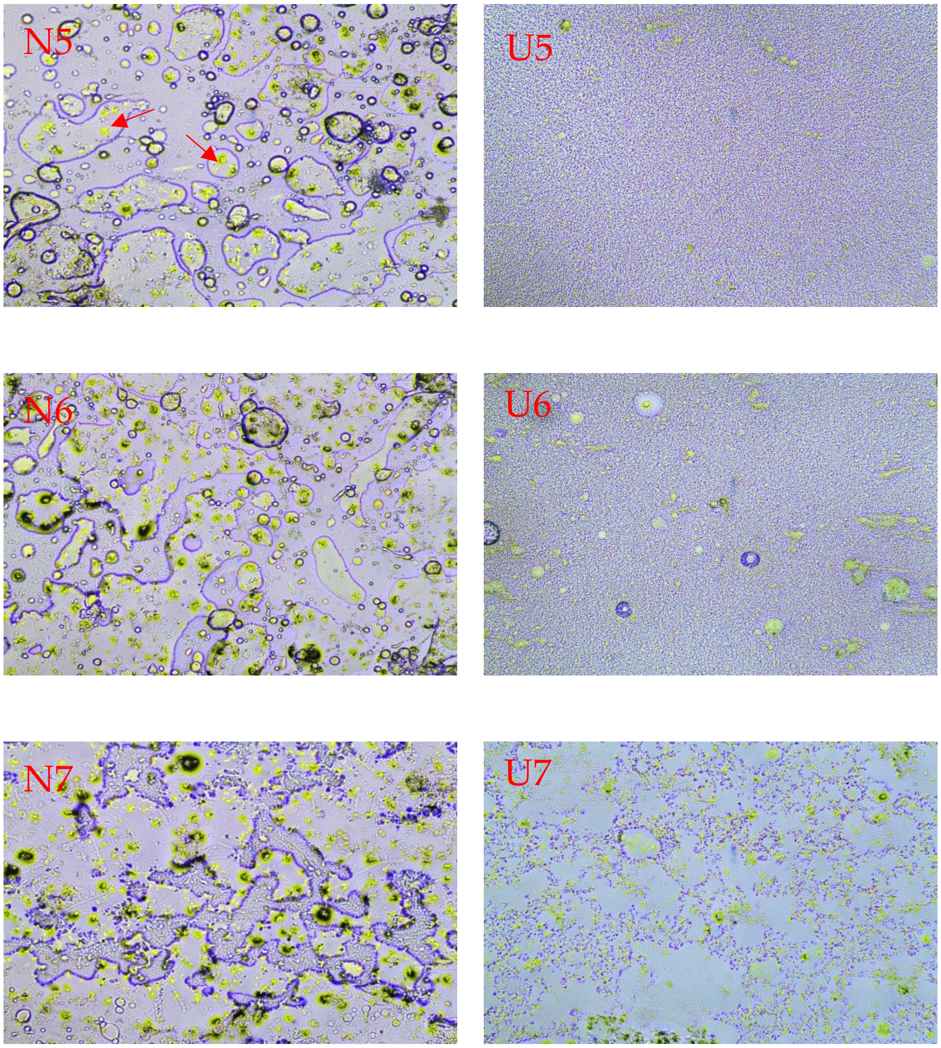

Figure S1. The distribution of oil droplets in emulsions as affected by high-intensity ultrasound emulsification. The red arrow points to the pork fat, and the red circle is the emulsion drop.
